# Red Flags: Clinical Signs for Identifying Autoimmune Encephalitis in Psychiatric Patients

**DOI:** 10.3389/fpsyt.2017.00025

**Published:** 2017-02-16

**Authors:** Julia Herken, Harald Prüss

**Affiliations:** ^1^Autoimmune Encephalopathies Group, German Center for Neurodegenerative Diseases (DZNE) Berlin, Berlin, Germany; ^2^Department of Neurology and Experimental Neurology, Charité – Universitätsmedizin Berlin, Berlin, Germany

**Keywords:** autoimmune encephalitis, schizophreniform syndrome, cerebrospinal fluid analysis, anti-neuronal autoantibodies, immunotherapy

## Abstract

Autoimmune mechanisms causing diverse psychiatric symptoms are increasingly recognized and brought about a paradigm shift in neuropsychiatry. Identification of underlying antibodies against neuronal ion channels or receptors led to the speculation that a number of patients go misdiagnosed with a primary psychiatric disease. However, there is no clear consensus which clinical signs in psychiatric patients should prompt further investigations including measurement of anti-neuronal autoantibodies. We therefore aimed to analyze the presenting symptoms in patients with autoimmune encephalitis and the time between symptom onset and initiation of antibody diagnostics. For this, we recruited 100 patients from the Charité Center for Autoimmune Encephalitis between May and October 2016, including all types of autoimmune encephalitides. Psychiatric abnormalities were the most common clinical symptoms and were the presenting sign in 60%. One-third of patients were initially hospitalized in a psychiatric ward. All patients positive for antibodies against the *N*-methyl-d-aspartate receptor showed behavioral changes, hallucinations, memory deficits, catatonia, or delusions. Patients positive for antibodies against other cell surface or intracellular antigens were often hospitalized with a psychosomatic diagnosis. The time between occurrence of first symptoms and antibody testing was often alarmingly prolonged. In patients with symptom onset between 2013 and 2016, the mean delay was 74 days, in cases diagnosed between 2007 and 2012 even 483 days, suggesting though that increased awareness of this novel disease group helped to expedite proper diagnosis and treatment. By analyzing the medical records in detail, we identified clinical signs that may help to assist in earlier diagnosis, including seizures, catatonia, autonomic instability, or hyperkinesia. Indeed, reanalyzing the whole cohort using these “red flags” led to a 58% reduction of time between symptom onset and diagnosis. We conclude that the timely diagnosis of an autoimmune psychiatric disease can be facilitated by use of the described clinical warning signs, likely enabling earlier immunotherapy and better prognosis. Also, the threshold for cerebrospinal fluid analysis and autoantibody testing should be low.

## Introduction

The growing number of newly described autoimmune encephalitides has drawn a remarkable link between immunology and psychiatry within the last several years (1–3). Since the pioneering discovery of *N*-methyl-d-aspartate receptor (NMDAR) autoantibodies (4), various further antibodies against receptors and ion channels were identified in patients with psychiatric abnormalities, such as against AMPA, GABA, glycine receptors, metabotropic glutamate receptor 5 (mGluR5), and dopamine-D2 receptors (Table 1), not only in humans (5). Patients are often first hospitalized in psychiatric departments before being transferred to a neurology ward (6, 7), stimulating the intriguing question of whether a subset of patients may go misdiagnosed with a primary psychiatric disease (1, 2, 8, 9). Recently, a high prevalence of cerebrospinal fluid (CSF) abnormalities including the detection of anti-neuronal autoantibodies has been observed in 54.4% of psychotic patients (10), highlighting their potential role in psychiatry and underlining the need for increased clinical and scientific awareness in order to not overlook treatable etiologies.

**Table 1:** **Classification of encephalitis groups in the present study and commonly associated clinical features**.

Encephalitis groups of the present study	Antibodies	Number of patients	Psychiatric symptoms	Additional symptoms	Typical patient
(A) NMDAR encephalitis (*n* = 53)	NMDA receptor	*n* = 53 (53%)	Psychosis, schizophreniform illness, catatonia, hallucinations, aggression	Epileptic seizures, dyskinesia, autonomic instability, speech dysfunction, decreased consciousness	Young women, association with ovarian teratomas

(B) Non-NMDAR cell surface antigens (*n* = 24)	Caspr2	*n* = 4 (4%)	Insomnia, panic attacks, schizophreniform illness, depression	Morvan syndrome, neuromyotonia, muscle spasms, fasciculations	Middle age or elderly patients, may be associated with thymoma
	
	LGI1	*n* = 14 (14%)	Amnesia, confusion, memory deficits, depression	Limbic encephalitis, faciobrachial dystonic seizures, hyponatremia	Middle age or elderly patients, male:female (2:1), may be associated with thymoma
	
	Metabotropic glutamate receptor 5	*n* = 2 (2%)	Behavioral changes, emotional instability, memory deficits	Limbic encephalitis, Ophelia syndrome	Young adults, may be associated with Hodgkin’s lymphoma
	
	Glycine receptor	*n* = 1 (1%)	Behavioral changes, schizophreniform syndrome	Stiff-person syndrome (SPS) or progressive encephalomyelitis with rigidity and myoclonus, hyperekplexia	middle age or elderly patients, may be associated with thymomas and lymphomas

(C) Antibodies against intracellular antigens (*n* = 23)	Synaptic antigens: anti-GAD antibodies	*n* = 9 (9%)	Schizophreniform illness, autism, attention-deficit/hyperactivity disorder	Limbic encephalitis, seizures, SPS, brainstem dysfunction, ataxia	Middle age or elderly patients, might be associated with small-cell lung cancer
	
	Onconeuronal antigens: anti-Yo, -Hu, -CV2, -Ri, -Ma2 antibodies	*n* = 14 (14%)	Behavioral changes	Limbic encephalitis, cerebellar degeneration, sensory neuropathy	Elderly patients, often with malignant tumors (small-cell lung carcinoma, Hu; testicular seminoma, Ma2)

*NMDAR, N-methyl-d-aspartate-receptor; LGI1, leucine-rich glioma inactivated 1; Caspr2, contactin associated protein 2*.

Antibody-mediated encephalitides can be categorized based on the presence of anti-neuronal antibodies targeting (i) neuronal cell surface antigens and (ii) intracellular antigens (11, 12). Autoantibodies directed to cell surface proteins are more frequently found in patients with psychiatric abnormalities, likely due to a suspected direct pathogenic effect (12–14). The demonstration of specific effects of NMDAR antibody-containing CSF *in vivo* convincingly substantiates the link between autoantibodies and the schizophreniform syndrome seen in these patients (15). Most recent work using CSF-derived human monoclonal NMDAR antibodies showed that the antibody is sufficient to change NMDAR expression and electrophysiology (16). Thus, the presence of this antibody alone represents a risk factor for neuropsychiatric symptoms, supporting the need for sufficiently aggressive immunotherapy in affected patients.

Such a clear causative role of autoantibodies on psychiatric symptoms has yet to be shown for further surface-directed antibodies. Nonetheless, psychotic symptoms are common in numerous other autoimmune encephalitides (Table 1). For example, patients with antibodies against the voltage-gated potassium channel complex (VGKCc) often present with hallucinations, depression, and memory deficits (13, 14, 17). Neuropsychiatric symptoms were found in 44% of VGKCc antibody-positive patients, occasionally treated for primary psychiatric diagnoses (14). Less well known, patients with antibodies against intracellular targets can also present with psychiatric symptoms (18).

The prognosis of autoimmune encephalitides largely depends on the rapid initiation of immunotherapy. Any delay in diagnosis causes costs and morbidity, while early immunotherapy results in substantial recovery in 70–80% of the patients (6, 19–23). This is especially striking considering the often severe course of the disease, sometimes requiring prolonged episodes of intensive care unit treatment and mechanical ventilation (6). Delayed recognition of the disease can also result in inadequate use of neuroleptics, which in patients with NMDAR encephalitis frequently worsens the symptoms, leading to the working diagnosis of a neuroleptic malignant syndrome (7).

We therefore aimed to retrospectively ascertain the time and frequency of delayed diagnosis of autoimmune encephalitides and asked whether specific clinical signs can assist in earlier recognition, antibody testing, and proper diagnosis of the disease. Indeed, a number of warning signs (“red flags”) can help to facilitate the timely diagnosis of an autoimmune psychiatric disease, likely enabling earlier immunotherapy and better prognosis.

## Materials and Methods

### Patient Selection

**Figure 1:**
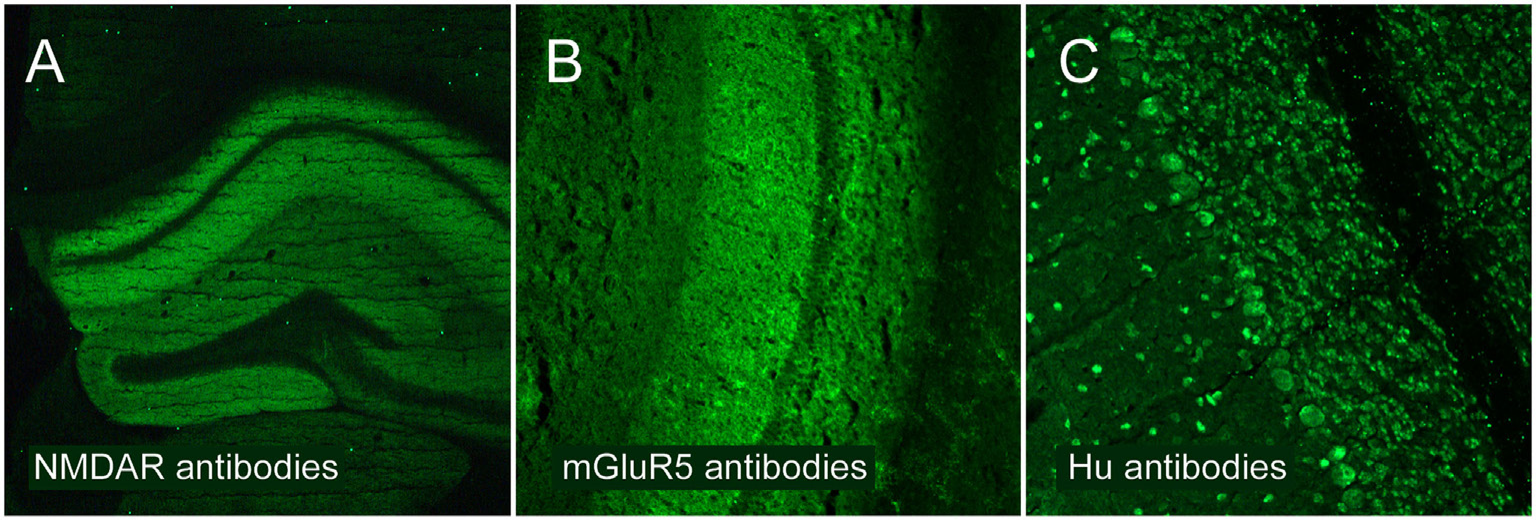
**Classification of encephalitis groups analyzed in the present study**. Underlying autoantibodies show different patterns of brain binding using immunofluorescence testing. **(A)** Patients with NMDAR encephalitis and high-level autoantibodies against the NR1 subunit of the NMDAR. **(B)** Patients with non-NMDAR antibodies targeting neuronal surfaces, such as antibodies against the metabotropic glutamate receptor 5 (mGluR5). **(C)** Patients with antibodies targeting intracellular epitopes, such as anti-Hu antibodies.

*N* = 100 patients with different forms of autoimmune encephalitides were recruited in the Charité Centre for Autoimmune Encephalitis from May to October 2016. Patients were grouped in three categories (Table 1):

(A) Anti-NMDAR encephalitis (*n* = 53), defined by a compatible clinical picture and positive IgG-NMDAR antibodies in the CSF (Figure 1A).
(B) Non-NMDAR surface antibodies (*n* = 24), including patients with antibodies against the neuronal cell surface antigens LGI1 (*n* = 14), CASPR2 (*n* = 4), mGluR5 (*n* = 2, Figure 1B), glycine receptor (*n* = 1) and against an unknown epitope determined on brain section immunofluorescence testing (*n* = 3).
(C) Antibodies against intracellular epitopes (*n* = 23), including patients with GAD antibodies (*n* = 9) or onconeuronal antibodies, such as Yo, Hu, Ri, or CV2 (*n* = 14, Figure 1C).

### Informed Consent

Written informed consent was received from participants at the Charité Department of Neurology or their representatives prior to inclusion in the study, and analyses were approved by the Charité University Hospital Institutional Review Board.

### Clinical Data Collection

Most patients were hospitalized in the Charité Department of Neurology during the disease course. Medical charts were retrospectively analyzed, and clinical and para-clinical information was collected during follow-up visits in the outpatient clinic or *via* email/telephone interviews. The following information was systematically retrieved from medical records: age, sex, date of disease onset, neurological and psychiatric symptoms during initial clinical presentation, psychiatric and neurological signs during follow-up, department of initial hospitalization, details of psychiatric hospitalization, symptoms that led to determination of antibodies, date of diagnosis, and time from first symptoms to diagnosis.

## Results

### Demographic Data

Median age in our cohort was 41 years (range 14–92 years) and 71% were female. Patients positive for NMDAR antibodies were younger (mean age 30 [14–57] years) and mainly women (91%). In contrast, patients with antibodies against non-NMDAR surface antigens were predominantly of male gender (67%) and older (mean age 53 [29–78] years). Patients positive for antibodies against intracellular proteins were predominantly female (65%), mean age was 56 (37–92) years.

### Initial Hospitalization in a Psychiatric Department

In order to estimate the overlapping symptoms with primary psychiatric disorders, we analyzed the frequency of patients initially hospitalized in a psychiatric department and the frequency of psychotic symptoms at first evaluation and during follow-up. *N* = 31 patients (31%) were initially hospitalized on a Psychiatry ward, commonly for psychotic or suspected psychosomatic symptoms. Almost two-thirds of all patients (*n* = 60; 60%) showed psychotic symptoms at the beginning of the disease, even if hospitalization was not required, 7% presented with psychosomatic symptoms.

Psychiatric symptoms were not equally distributed across the three encephalitis groups. All patients with NMDAR antibodies (*n* = 53) showed psychotic symptoms. In patients positive for antibodies against other neuronal surface or intracellular antigens, psychosomatic symptoms were common at presentation: 8/24 in the non-NMDAR group (33%), 5/23 in the intracellular antigens group (22%). However, psychotic symptoms did also occur: 6/24 in the non-NMDAR group (25%), 4/23 in the intracellular group (17%). Of the NMDAR antibody-positive patients, 21/53 (40%) were seen by a psychiatrist at first evaluation, while this was the case for only one patient positive for intracellular protein antibodies (4%).

### Initial Symptoms

The frequency of first clinical signs was again not equally distributed between the encephalitis groups (Table 2). Patients positive for NMDAR antibodies typically presented with psychiatric symptoms and either developed a spectrum of neurological abnormalities, such as seizures, movement, or speech disorders, or already showed them at first evaluation. Their initial psychiatric symptoms were acute behavioral changes (*n* = 46; 87%), hallucinations (*n* = 23; 43%), paranoid delusions (*n* = 13; 26%), and memory deficits, especially short-term memory loss (*n* = 11; 21%). Also, mutism (*n* = 8; 15%), catatonia (*n* = 10; 19%), and depressive symptoms (*n* = 10; 19%) were commonly seen at presentation. One young woman got initially hospitalized with the clinical picture of anorexia. First symptoms in some patient were neurological, consisting of epileptic seizures (*n* = 10; 19%), speech dysfunction such as pressured speech and verbal reduction (*n* = 10; 19%), dyskinesia (*n* = 7; 13%), and headache (*n* = 9; 17%).

**Table 2:** **Presenting clinical symptoms in all 100 patients**.

Initial signs and symptoms	All patients (100)	NMDAR (53)	Non-NMDAR (24)	Intracellular antigens (23)
**Psychiatric**				
Acute behavioral changes	56 (56%)	46 (87%)	7 (29%)	3 (13%)
Hallucinations (visual, auditory)	25 (25%)	23 (43%)	1 (4%)	
Memory deficits (retro- and anterograde amnesia)	22 (22%)	11 (21%)	8 (33%)	4 (17%)
Confusion/aggression	18 (18%)	11 (21%)	6 (25%)	1 (4%)
Paranoid delusions	17 (17%)	13 (26%)	2 (8%)	1 (4%)
Depressed mood	13 (13%)	10 (19%)	4 (16%)	1 (4%)
Catatonia	10 (10%)	10 (19%)		
Mutism	8 (8%)	8 (15%)		
Anorexia	1 (1%)	1 (2%)		
Any of the above symptoms	65 (65%)	53 (100%)	14 (58%)	7 (30%)
**Neurological**				
Sensorimotor deficits	30 (30%)	8 (15%)	7 (29%)	13 (57%)
Seizures		10 (19%)	2 (8%)	5
Generalized tonic-clonic	13 (13%)	9 (17%)	1 (4%)	3 (13%)
Focal	4 (4%)	1 (2%)	1 (4%)	2 (9%)
Faciobrachial dystonic seizures	7 (7%)		7 (29%)	
Speech dysfunction (pressured speech, verbal reduction)	15 (15%)	10 (19%)	4 (16%)	
Movement disorders	11 (11%)	7 (13%)	1 (4%)	3 (13%)
Headache	12 (12%)	9 (17%)	1 (4%)	2 (9%)
Reduced levels of consciousness	7 (7%)	5 (9%)	2 (8%)	
Paralysis	7 (7%)	4 (8%)	1 (4%)	2 (9%)
Cerebellar ataxia	10 (10%)	1 (2%)	3 (12%)	7 (30%)
Diplopia	7 (7%)	3 (6%)		4 (17%)
Any of the above symptoms	67 (67%)	39 (74%)	20 (83%)	20 (87%)

Patients of the non-NMDAR group presented also with psychiatric symptoms in most cases, such as acute behavioral changes (*n* = 7; 29%), aggression/confusion (*n* = 6; 25%), or memory deficits (*n* = 8; 33%). Hallucinations and paranoid delusions were also seen (Table 2). The neurological symptoms of this group were more characteristic and included faciobrachial dystonic seizures (FBDS, in patients with LGI1 antibodies) (*n* = 7; 29%) and sensorimotor deficits (*n* = 7; 29%).

Patients positive for intracellular epitope antibodies presented less frequently with psychiatric symptoms, including acute behavioral changes and memory deficits. The majority of symptoms in this group were neurological, such as sensorimotor deficits (*n* = 13; 57%), cerebellar ataxia (*n* = 7; 30%), movement disorders (*n* = 3; 13%), and generalized tonic-clonic seizures (*n* = 3; 13%).

In most patients of all three groups, both psychiatric and neurological symptoms occurred during the first month of disease. Interestingly, *n* = 13 (13%) of all patients presented with a depressed mood, in four cases leading to the diagnosis of major depression. Appearance of additional neurological symptoms led to reclassification of diagnosis.

### Which Clinical Features Led to Examination of Autoantibodies?

We next determined which clinical symptoms, routine laboratory findings, or imaging abnormalities triggered the testing for autoantibodies in all 100 patients, the results of which finally allowed the firm diagnosis of autoimmune encephalitis (Table 3). Indeed, several clinical constellations of neurological and psychiatric symptoms were more common than others to stimulate antibody testing. We semi-quantitatively classified these constellations as “yellow flags” and “red flags,” depending on their power to predict the presence of autoantibodies in such patients (Table 4).

**Table 3:** **Clinical symptoms and constellations that led to the determination of anti-neuronal antibodies in all 100 patients**.

Symptoms	All patients (100)	NMDAR (53)	Non-NMDAR (24)	Intracellular antigens (23)
Epileptic seizures	14 (14%)	10 (19%)	2 (8%)	2 (8%)
Cerebrospinal fluid (CSF) abnormalities^a^ and absent evidence for infectious encephalitis	13 (13%)	12 (27%)	1 (4%)	
Abnormal postures or movements	4 (4%)	4 (7%)		
Reduced levels of consciousness	4 (4%)	4 (7%)		
Aphasia or dysarthria	3 (3%)	3 (6%)		
Lack of improvement with antipsychotics	5 (5%)	4 (7%)	1 (4%)	
Autonomic instability	2 (2%)	2 (4%)		
Suspicious MRI or EEG findings	10 (10%)	3 (6%)	5 (20%)	2 (8%)
Steroid-responsive autoimmune thyroiditis	3 (3%)	2 (4%)		1 (4%)
Lack of improvement with antiepileptic medication	2 (2%)	1 (2%)	1 (4%)	
Focal neurological deficits	3 (3%)	1 (2%)	1 (4%)	1 (4%)
Sensory deficits	3 (3%)	1 (2%)	2 (8%)	
Rapidly progressing psychosis	4 (4%)	1 (2%)	2 (8%)	1 (4%)
Suggested by patients or families	3 (3%)	3 (6%)		
Positive effect of *ex juvantibus* immunotherapy	2 (2%)		1 (4%)	1 (4%)
Faciobrachial dystonic seizures	3 (3%)		3 (12%)	
Neuromyotonia	1 (1%)		1 (4%)	
Cerebellar ataxia	8 (8%)		2 (8%)	6 (26%)
Hyponatremia	2 (2%)		2 (8%)	
Paresthesia or malignant tumor^b^	7 (7%)			7 (30%)

*^a^Increased white blood cell count or CSF-specific oligoclonal bands*.

*^b^Small-cell lung cancer, testicular seminoma*.

**Table 4:** **Warning signs pointing to an autoimmune etiology in new-onset psychosis**.

**Yellow flags** 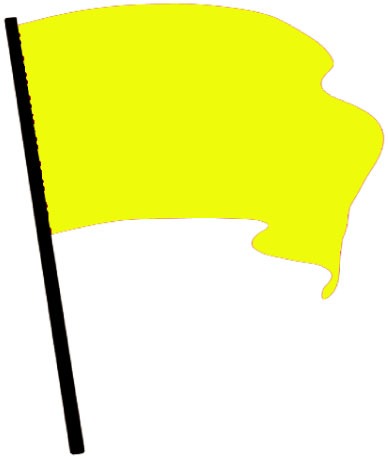	Decreased levels of consciousness Abnormal postures or movements (orofacial, limb dyskinesia) Autonomic instability Focal neurological deficits Aphasia or dysarthria Rapid progression of psychosis (despite therapy) Hyponatremia Catatonia Headache Other autoimmune diseases (e.g., thyroiditis)
**Red flags** 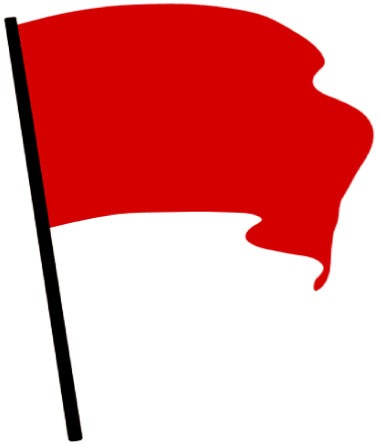	Cerebrospinal fluid (CSF) lymphocytic pleocytosis or CSF-specific oligoclonal bands without evidence for infection Epileptic seizures Faciobrachial dystonic seizures Suspected malignant neuroleptic syndrome MRI abnormalities (mesiotemporal hyperintensities, atrophy pattern) EEG abnormalities (slowing, epileptic activity or extreme delta brush)

*“Red flag” criteria should always prompt determination of anti-neuronal autoantibodies in psychiatric patients. “Yellow flag” criteria should raise suspicion of an autoimmune etiology and include autoimmune encephalitis in the differential diagnoses, in either case if several findings are present*.

In the NMDAR encephalitis group, viral encephalitis was a common working diagnosis, often suggested by the clinical picture, acute neurological changes, and CSF pleocytosis. NMDAR autoantibody testing was often initiated once the search for a viral or bacterial pathogen remained negative (*n* = 12; 27%). In all three groups, the occurrence of epileptic seizures frequently initiated CSF investigation including determination of antibodies (*n* = 14; 14%). Suspicious MRI and EEG were another reason for antibody testing, in particular in patients with non-NMDAR surface antibodies (*n* = 5; 20%), but much less in NMDAR antibody-positive patients (*n* = 3; 6%). Patients were frequently transferred from a psychiatric to a neurological ward at this point. Similarly, in patients hospitalized for a schizophreniform syndrome, detection of abnormal neurological signs resulted in antibody testing. These deficits included decreased levels of consciousness (*n* = 4; 7%), abnormal postures or movements (*n* = 4; 7%), and aphasia or dysarthria (*n* = 3; 6%) in patients of the NMDAR encephalitis group. Focal neurological signs were the trigger for antibody testing in one patient each of the NMDAR (2%), non-NMDAR surface antibody (4%), and intracellular epitope antibody (4%) groups (Table 3).

Non-NMDAR antibodies testing was performed in several cases because of the occurrence of FBDS (*n* = 3; 12%), sensory deficits (*n* = 2; 8%), or the detection of hyponatremia in the context of unexplained neuropsychiatric symptoms (*n* = 2; 8%). In two patients with non-NMDAR surface antibodies, antibody testing was initiated because of a rapidly progressing psychosis (*n* = 2; 8%). A common reason to test for antibodies against intracellular epitopes was the occurrence of paresthesia in the context of a malignant tumor (*n* = 7; 30%) or clinical deficits resulting from cerebellar symptoms (*n* = 6; 26%).

We further identified seven cases in which the lack of clinical improvement after antipsychotic (*n* = 5; 5%) or antiepileptic therapy (*n* = 2; 2%) led to the suspicion of an autoimmune encephalitis. Another two patients with psychotic symptoms and cognitive impairment had the working diagnosis of steroid-responsive encephalopathy with autoimmune thyroiditis (SREAT), which triggered antibody testing that resulted in positive NMDAR (*n* = 2; 4%) and onconeuronal (*n* = 1; 4%) antibodies (Table 3). Finally, in one case, the patient’s family suggested the diagnosis of autoimmune encephalitis after internet research, prompting the testing of NMDAR antibodies which returned positive.

Taken together, several clinical symptoms and abnormalities repeatedly led to antibody testing, bringing about the correct diagnosis of autoimmune encephalitis. We consider these warning signs as “red flags” (Table 4) which might facilitate earlier diagnosis of autoimmunity in psychiatric symptoms.

### Time from First Symptom to Diagnosis

**Figure 2:**
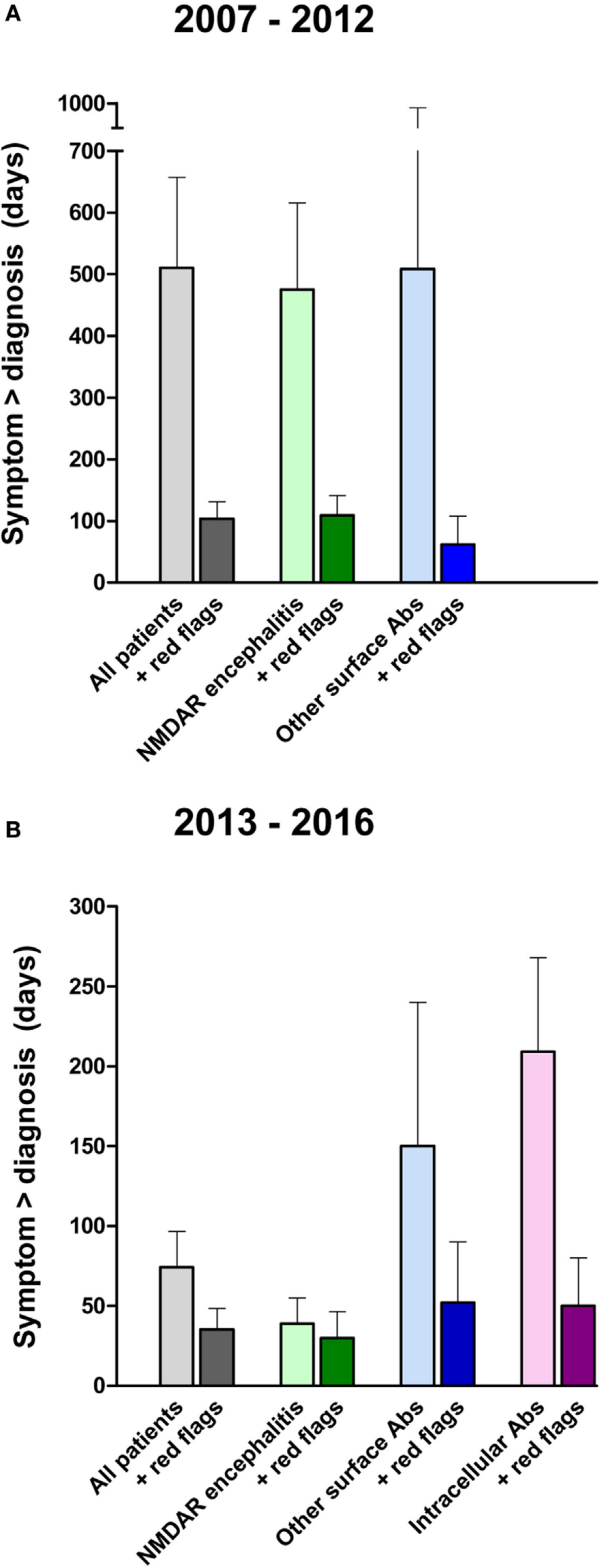
**Time between onset of clinical symptoms and diagnosis of antibody-associated encephalitis**. Comparing patients with disease onset between 2007 and 2012 **(A)** versus 2013 to 2016 **(B)**, the delay from symptom onset to the diagnosis of autoimmune encephalitis (light colors) has been reduced within the last years, likely due to increased awareness (please note the different *y*-axes). Applying the “red flag” criteria to the same patients by reanalyzing medical records resulted in a marked hypothetical reduction of the delay until antibody testing and encephalitis diagnosis (dark colors).

Given that the prognosis in patients with autoimmune encephalitis depends on the rapid initiation of immunotherapy, we next analyzed the time between symptom onset and diagnosis. For this, patients who were treated primarily in a psychiatry department (*n* = 35) were divided in two groups. In the first group, symptoms started between 2007 and 2012. Here, the delay was very prolonged with a mean time of 483 days (Figure 2A). In the second group with symptom onset between 2013 and 2016, the mean time between disease onset and diagnosis was 74 days (Figure 2B). The reduction was obvious in both groups for which data were available, namely the NMDAR encephalitis (reduction from 475 to 40 days) and non-NMDAR antibody group (reduction from 509 to 150 days). It seems likely that increased awareness of this new disease group after 2012 and a lower threshold for antibody testing in clinical routine helped to markedly reduce the delay, even though there is an obvious need and opportunity for further improvement.

### Earlier Diagnosis of Autoimmune Encephalitis in Psychiatric Patients Using the “Red Flags”

Having established warning signs (“yellow flags” and “red flags”) that may guide clinicians in the indication for autoantibody testing in patients with different autoimmune encephalitides (Table 4), we then retrospectively applied these criteria to our cohort of encephalitis patients hospitalized in a psychiatric ward. In this way, we aimed to estimate the potential reduction in delay between symptom onset and diagnosis of autoimmune encephalitis. Indeed, reanalysis of the medical records showed that most patients had well-documented evidence of “yellow flag” and “red flag” criteria in their medical records, long before an autoimmune etiology and antibody testing was considered. As a typical example, a patient with a schizophreniform syndrome developed catatonia and autonomic instability (both are “yellow flags”) 4 weeks after the symptom onset, but only an epileptic seizure 10 weeks after symptom onset prompted autoantibody testing and revealed positive NMDAR antibodies. We then calculated the time from symptom onset to diagnosis, hypothetically assuming that the first documentation of a “red flag” in the medical chart would have resulted in the determination of autoantibodies. In this example, using the “yellow flag” and “red flag” criteria reduced the delay from symptom onset to diagnosis from 10 to 4 weeks.

Indeed, the analysis of our cohort showed a marked reduction in the time until diagnosis (Figure 2). For the more recent patients with symptom onset between 2013 and 2016, a reduction of 58% from 74 to 31 days was detectable. In detail, time between appearance of first symptoms and final diagnosis was reduced from 40 to 10 days (75%) in patients with NMDAR encephalitis, 150 to 52 days (65%) in patients with non-NMDAR surface antibodies, and 209 to 50 days (76%) in patients with antibodies against intracellular epitopes (Figure 2B).

## Discussion

In accordance with recent publications (7, 8, 24), our results confirm that a broad spectrum of psychiatric symptoms frequently are the first complaints in patients with autoimmune encephalitis. While psychosis typically led to hospitalization of patients with NMDAR encephalitis, a psychosomatic disorder was often suspected in patients with surface non-NMDAR and intracellular epitope antibodies. The “psychosomatic” symptoms included, for example, FBDS in LGI1 antibody-positive patients, muscle spasms, and fasciculations in Caspr2 antibody-positive patients or sensory deficits in patients with onconeuronal antibodies. Interestingly, most patients in all three encephalitis groups showed additional neurological symptoms during the first month of disease.

Analysis of the present cohort of 100 encephalitis patients showed that several clinical symptoms or laboratory findings eventually led to the suspicion of an autoimmune etiology and the determination of autoantibodies. These “yellow flags” and “red flags” are summarized in Table 4, classified based on their predictive value to point to an underlying autoimmune encephalitis in the clinical workup of patients with psychiatric abnormalities. Given that systematic controlled trials and systematic reviews of cohort or case-control studies are lacking due to the novelty of this field and the relative rarity of autoimmune encephalitides, this case series analysis can only represent level 4 of evidence. Generally, some constellations are very typical for a given form of encephalitis, e.g., the presence of new-onset psychosis in young women with ovarian teratomas indicating NMDAR encephalitis or the combination of amnesia, hyponatremia, and the pathognomonic FBDS (brief repetitive stereotyped movements predominantly affecting the arm and ipsilateral face) indicating LGI1 antibody encephalitis. Clearly, typical features can be absent and delay the proper diagnosis (11, 25–27). Also, future work will likely add further or modify the proposed criteria.

The most common triggers for autoantibody diagnostic were CSF abnormalities in the absence of an infectious disease. The symptom overlap with viral encephalitis is remarkable regarding neurological and psychiatric changes (28, 29), suggesting that autoantibodies should always be determined, at the latest if virus diagnostic (using PCR) remains negative. CSF is abnormal in almost all patients with NMDAR encephalitis during the disease course (11, 24), underlining the relevance of routine CSF testing in psychiatric patients. This is also valid for the other forms of encephalitis, although patients with LGI1 antibodies have a lower frequency of CSF pleocytosis (41%) or elevated protein (47%) and rarely have intrathecal LGI1 antibody synthesis (25).

The occurrence of epileptic seizures in a psychotic patient was another common reason to reassess the working diagnosis of a primary psychiatric disease and test for antibodies. EEG changes not explained by medication are almost always present in autoimmune encephalitis. The alterations are rarely specific, showing focal or diffuse slow activity frequently associated with one or several foci of epileptic activity, eventually revealing subclinical seizures (27). However, the pattern referred to as “extreme delta brush” in NMDAR encephalitis is quite disease-specific (30). Suspicious MRI findings led to the correct diagnosis in relatively few cases in the present cohort (10%), which is likely explained by the fact that brain MRIs are unremarkable in more than 50% of patients with NMDAR encephalitis (11, 23, 28). If present, however, MRI abnormalities should always prompt autoantibody investigation, even though other diseases might cause similar imaging changes, such as gliomas (25, 28, 31).

Lejuste et al. observed a very high rate of patients with NMDAR encephalitis in which intolerance to antipsychotic drugs led to transfer to a Neurology department or intensive care unit (7). In line with their findings, the combination of autonomic instability and increased creatine kinase levels after neuroleptic therapy in several cases led to the suspicion of a malignant neuroleptic syndrome. Therefore, we included progression under antipsychotic therapy, suspected malignant neuroleptic syndrome and autonomic instability to the “red flag” criteria (Table 4). Finally, the presence of an autoimmune thyroiditis together with psychotic symptoms and cognitive impairment resulted in antibody investigation in three cases in the present cohort. It was shown recently that serum thyroid antibodies were elevated in 24.7% of 180 psychotic patients (10). Beneficial effects from steroids suggest the less well-defined constellation of SREAT (32). However, occurrence of specific brain-directed antibodies in our cohort (e.g., NMDAR antibodies) support the idea that SREAT represents increased susceptibility to autoimmunity, rather than that antithyroid antibodies are directly pathogenic. Findings of elevated thyroid peroxidase and thyroglobulin antibodies in psychotic patients should nonetheless raise suspicion and guide autoantibody testing.

Apart from the clinical application of the here proposed criteria, the present study reinforces the recent discussion that autoantibodies may participate in the development of psychiatric disorders, such as schizophrenia, in greater extend than previously assumed. For example, the reduction of NMDAR-specific currents and consecutively impaired glutamatergic neurotransmission is well known under the NMDAR hypofunctionality hypothesis of schizophrenia (33). In parallel, synaptic and extrasynaptic reduction of NMDAR by autoantibodies in NMDAR encephalitis leads to the typical schizophreniform symptoms seen in these patients (34). While internalization of NMDAR after contact with autoantibodies has been established as an important disease mechanism (16, 35), further pathologies are likely to happen in parallel, such as chemokine transfer from immune cells to NMDAR-bearing neurons *via* volume transmission (36). It seems that these novel synaptic and extrasynaptic autoimmune disorders have brought about a paradigm shift in neuropsychiatry, and further research is urgently needed to clarify the detailed mechanisms of how autoimmunity and inflammation cause or modify neuropsychiatric diseases.

An important finding of our study was the alarmingly long delay between first symptoms and the final diagnosis of autoimmune encephalitis in many cases. It is known from the literature that patients with autoimmune encephalitis have often been misdiagnosed with a sole psychiatric disease despite the presence of neurological comorbidities (7). We could show here that the identification of encephalitis patients occurred much faster in more recent cases (2013–2016) compared to earlier patients, likely due to increased awareness of this novel disease group. The data collectively suggest that continuing increase in disease awareness will lead to further shortening of the time until diagnosis. This is needed as early and sufficiently aggressive immunotherapy is required for a better prognosis (22, 23, 25, 26). Using the here proposed “yellow flag” and “red flag” criteria will likely facilitate the timely diagnosis of an autoimmune psychiatric disease, as demonstrated by the hypothetical reanalysis of our cohort for the presence of such clinical signs. Finally, we conclude that CSF analysis should become clinical routine in patients with new-onset psychosis for several reasons. First, CSF abnormalities were the major indicator for an autoimmune encephalitis in psychotic patients. Second, some antibodies including NMDAR antibodies can be present in CSF only and would therefore be overlooked in serum (37). Third, recent data suggest that the rate of CSF abnormalities can be >50%, thus being much higher than previously thought and an important step to identify patients with treatable etiologies (10). Taken together, the threshold for CSF analysis and autoantibody testing should be low, in particular, when “red flags” are present.

## Author Contributions

JH and HP initiated the study and conducted the data analyses, wrote the paper, performed the data collection, read and approved the final version of this manuscript.

## Conflict of Interest Statement

The authors declare that the research was conducted in the absence of any commercial or financial relationships that could be construed as a potential conflict of interest.
